# The Immunologic Downsides Associated with the Powerful Translation of Current COVID-19 Vaccine mRNA Can Be Overcome by Mucosal Vaccines

**DOI:** 10.3390/vaccines12111281

**Published:** 2024-11-14

**Authors:** Maurizio Federico

**Affiliations:** National Center for Global Health, Istituto Superiore di Sanità, 00161 Rome, Italy; maurizio.federico@iss.it

**Keywords:** COVID-19 mRNA vaccines, SARS-CoV-2 Spike, mucosal vaccines, ACE-2, autoimmunity

## Abstract

The action of mRNA-based vaccines requires the expression of the antigen in cells targeted by lipid nanoparticle–mRNA complexes. When the vaccine antigen is not fully retained by the producer cells, its local and systemic diffusion can have consequences depending on both the levels of antigen expression and its biological activity. A peculiarity of mRNA-based COVID-19 vaccines is the extraordinarily high amounts of the Spike antigen expressed by the target cells. In addition, vaccine Spike can be shed and bind to ACE-2 cell receptors, thereby inducing responses of pathogenetic significance including the release of soluble factors which, in turn, can dysregulate key immunologic processes. Moreover, the circulatory immune responses triggered by the vaccine Spike is quite powerful, and can lead to effective anti-Spike antibody cross-binding, as well as to the emergence of both auto- and anti-idiotype antibodies. In this paper, the immunologic downsides of the strong efficiency of the translation of the mRNA associated with COVID-19 vaccines are discussed together with the arguments supporting the idea that most of them can be avoided with the advent of next-generation, mucosal COVID-19 vaccines.

## 1. Introduction

COVID-19 mRNA-based vaccines have been distributed to many people in both their original and current updated versions. Furthermore, mRNA technology is the basis of additional experimental vaccines as well as the latest generation of anticancer immunotherapies. Hence, it is mandatory to identify, monitor, and deeply analyze the most relevant unexpected events that this technology can produce in humans, even if these occur rarely.

Several features distinguish the mRNA-based COVID-19 vaccines from the “traditional” ones based on attenuated/inactivated viruses, subunit products, or recombinant products, which have been so useful for the elimination/containment of several infectious diseases. First, the vaccine formulation comprises lipidic nanoparticles (LNPs) complexed with mRNA molecules produced through the in vitro transcription process. Second, the immunogen is not part of the vaccine formulation, but it is expected to be synthesized by cells internalizing the mRNA/LNP complexes. This evidence justifies the more appropriate definition of prodrug (intended as a pharmacologically inactive substance that is converted in the body into a pharmacologically active drug) rather than vaccine [1]. Third, the immunogen (i.e., the viral protein Spike) is synthesized by target cells at very high levels and persists over time [2]. Fourth, the immunogen recognizes, binds, and activates a widespread signaling cell receptor, i.e., the angiotensin-converting enzyme (ACE)-2, and is stabilized in its prefusion conformation through two consecutive mutations to proline at amino acid positions 986 and 987, which do not negatively impact ACE-2 binding/activation. Hence, the abundance, diffusion, persistency, biologic activity, and stability of the immunogen are key points distinguishing mRNA-based COVID-19 vaccines.

In this paper, the most relevant consequences of both the overproduction of the Spike antigen after mRNA-based COVID-19 vaccination and the rather potent circulatory immune response evoked are discussed. A comprehensive picture of all possible concerns would be of major utility for the development of safer and more targeted vaccines against SARS-CoV-2 and other airborne infectious agents. Among these, mucosal vaccines deserve some consideration given their action at the virus port of entry and the lack of unwanted systemic effects.

## 2. High and Persistent Levels of Circulating Spike After Vaccination

mRNA/lipidic nanoparticle (LNP) complexes can enter any cell type. Injection into the deltoid muscle favors their entry into muscle cells; however, the moderate inflammation induced by some lipidic components [3] can attract professional antigen-presenting cells (APCs) to the injection site. APCs can ingest the LNPs, undergo activation, and migrate to the lymph nodes [4]. Moreover, unquantifiable amounts of injected mRNA/LNP complexes escape cell internalization at the site of injection, thus entering into circulation. Consistently, biodistribution studies carried out by a manufacturer of COVID-19 mRNA vaccines highlighted the potential diffusion of intramuscularly injected LNPs in almost all tissues [5].

Both mRNA and vaccine Spike persist in the body for a long time after vaccination. A study carried out on autoptic samples from patients after COVID-19 vaccination demonstrated the persistence of the vaccine mRNA in bilateral axillary lymph nodes up to 30 days after vaccination [6]. Notably, vaccine mRNA was also found in both the heart ventricles up to 20 days after injection, and its presence correlated with myocardial injuries associated with an abnormally high number of myocardial macrophages. In another study, vaccine mRNA was found up to 60 days after the second dose in biopsies from ipsilateral axillary lymph nodes [2].

Part of the intracellularly expressed Spike remains exposed on the plasma membrane of target cells in its trimeric form, while a consistent fraction of it can shed and circulate. Accordingly, a median of 47 pg/mL of free Spike has been measured in the plasma of vaccinees 1–2 days after injection, with peaks of 174 pg/mL [2]. These levels of Spike in plasma appear surprisingly high, ranging, for instance, in the concentrations of inflammatory cytokines detected in subjects with acute systemic inflammation [7]. This evidence is of particular relevance given the high affinity of Spike for ACE-2, i.e., a widespread cell receptor involved in several key physiologic processes.

## 3. ACE-2: Summary of Functions, Distribution, and Signaling upon Spike Binding

ACE-2 is an 805-amino-acid-long, type I transmembrane protein with an extracellular glycosylated N-terminal region containing the carboxypeptidase domain whose function is removing single amino acids from the C-terminus of its substrates. ACE-2 is a key regulator of the renin–angiotensin–aldosterone system, which controls blood pressure. It catalyzes the conversion of angiotensin I, a decapeptide, to angiotensin 1–9, which can be converted to smaller, vasodilator angiotensin peptides (e.g., angiotensin 1–7) by ACE in the lungs. ACE-2 binds angiotensin II also, i.e., an octapeptide generated by ACE-driven cleavage of angiotensin I, to produce the vasodilator angiotensin 1–7. ACE-2 is also involved in the production of bradykinins, i.e., a group of peptides with potent vasodilator effects [8].

ACE-2 is expressed by a wide variety of cells including enterocytes, cardiomyocytes, renal tubules, vasculature, and ductal cells. Conversely, ACE-2 expression in respiratory tissues is limited to a small number of specialized cell types, i.e., type II alveolar cells and alveolar macrophages [9].

The interaction between ACE-2 and angiotensin II induces various signaling pathways ultimately leading to the release of several cytokines including IL-6, TNF-α, and TGF-β [10]. Notably, the effects of the interaction of ACE-2 with Spike recapitulate those described for it binding with its natural ligands [11]. In particular, in vascular endothelial cells, natural Spike generates a block of mitochondrial functions [12]; meanwhile, switching integrin ⍺5β1-dependent signaling leads to nuclear translocation of NF-κB. These events ultimately induce the expression of VCAM-1, ICAM-1, coagulation factors, and the release of TNFα, IL-1β, and IL-6 inflammatory cytokines [13]. Similar activation mechanisms have been reported for both macrophages and dendritic cells [14,15]. Importantly, natural Spike induces in both epithelial and endothelial cells the release of pleiotropic TGF-β cytokine [16].

## 4. The SARS-CoV-2 Spike/ACE-2/TGF-β Axis in the Anti-Tumor Immune Surveillance and the Epithelial to Mesenchymal Transition

The binding of Spike with ACE-2 produces profound alterations in intracellular signaling with the activation of transcription factors and the release of several soluble factors. In particular, human vascular endothelial cells treated with Spike have been found to release both TGF-β1 and TGF-β2 [17], consistent with previous “in vivo” evidence suggesting a key role of TGF-β in COVID-19 pathogenesis [18,19].

**Figure 1:**
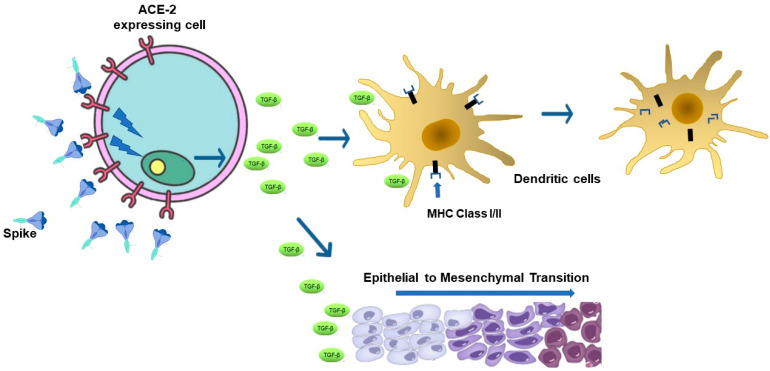
Bystander effects of Spike/ACE-2 binding. Free SARS-CoV-2 Spike protein binds ACE-2-expressing cells, thereby inducing intracellular signaling, leading to the release of soluble factors. Among these, TGF-β is known to downregulate the antigen-presenting activity in APCs through MHC Class I/II downregulation. TGF-β is also a major driver of the epithelial-to-mesenchymal transition that is the basis of the development of both solid tumors and metastasis.

TGF-β, with its three isoforms, i.e., -β1 to -β3, is a key regulator of the adaptive immune response [20], acting, for instance, as an inhibitor of the antigen-presenting activity in dendritic cells (DCs) through the downregulation of major histocompatibility complex (MHC) molecules [21,22] (Figure 1). It also reduces the expression of IL-12 and co-stimulatory molecules such as CD40 in macrophages and CD80, CD83, and CD86 in DCs, as part of the regulatory mechanisms of APC-mediated immune cell activation [23,24].

TGF-β can also interfere with the immune surveillance mechanisms controlling tumor cell growth. For instance, TGF-β can induce the polarization of macrophages from M1 (marked by the release of inflammatory cytokines such as IL-1β, IFN-γ, TNF-α, IL-12, and IL-18) to M2 macrophages, secreting anti-inflammatory cytokines like IL-1ra and IL-10, and characterized by multiple immunosuppressive properties of the tumor microenvironment [25]. On the other hand, TGF-β is a major driver of the epithelial-to-mesenchymal transition (EMT) [26], which is the basis of the development of both solid tumors and metastasis. In this scenario, consistent results from the experimental work of two research groups raised the hypothesis that natural Spike can contribute to the EMT (Figure 1). In detail, Lai and colleagues provided evidence that TGF-β-related signaling is part of the mechanism underlying the acquisition of a mesenchymal-like phenotype of Spike-expressing human breast cancer cells. Most importantly, they demonstrated that the number of lung metastases in mice inoculated with Spike-expressing 4T1 breast cancer cells increased compared to that induced by parental cells [27,28]. Ciszewski and colleagues observed that the treatment with recombinant, wild-type Spike of both HUVECs and HMEC-1 human endothelial cells induces the release of TGF-β associated with cell trans-differentiation. By investigating the underlying mechanism of action, they proved the involvement of the ACE-2/TGF-β/MRTF (myocardin-related transcription factor)-β axis in the observed EMT. Finally, the contribution of TGF-β in the Spike-related EMT was further corroborated by the demonstration that Spike-treated human endothelial cells failed to trans-differentiate in the presence of anti-TGF-β antibodies [17].

The results from these studies pose the question as to whether Spike can contribute to the EMT in humans. Even if no clinical data describing events associated with these pathological immune responses are available so far, the potential implications in terms of the safety of COVID-19 vaccines seem to manifest also considering the evidence that mRNA/LNPs can enter any kind of cell. For instance, the unfortunate entry of mRNA/LNP complexes into already emerged tumor cells may reproduce the conditions described by Lai and colleagues, thus representing a hazard in terms of the formation of metastases. On the other hand, pathogenetic bystander effects can be induced through the local production of high concentrations of Spike by normal cells targeted by the mRNA/LNPs and located in the vicinity of tumor cells, as described by Ciszewski and coll. For these reasons, expanding the studies to additional cell systems as well as to appropriate “in vivo” models appears mandatory considering the possibility that mRNA/LNP complexes circulate in the body after vaccination.

## 5. mRNA COVID-19 Vaccine-Induced Unspecific Immunity: Antibody Cross-Binding, Autoantibodies, Anti-Idiotype Antibodies, and Ribosomal Frameshifting

The high levels of vaccine Spike produced after injection are associated with an extraordinarily potent circulatory immune response, with the production of high titers of anti-Spike antibodies. On the one hand, this outcome is considered an advantage in terms of antiviral protection; on the other hand, however, such powerful immunogenicity can be associated with relevant unwanted effects typically emerging in the presence of both high and persistent antigenic stimuli. These include the substantial binding of anti-Spike antibodies cross-reacting with “self” antigens with the induction of non-physiologic/pathogenetic processes, the emergence of autoantibodies, and the generation of anti-idiotype antibodies. These events have been correlated with the emergence in vaccinees of pathologies like thrombocytopenia, myocarditis, various disturbances to the menstrual cycle, the re-emergence of latent infections, and post-COVID vaccine syndrome (PCVS).

Cross-reacting antibodies bind heterologous targets through the mechanism of molecular mimicry. Most likely, pathogenetic effects can be produced when sufficient amounts of them bind unspecific molecular targets acting in relevant biological processes. Through a computationally investigated analysis of the molecular mimicry between Spike and known human epitopes, it was reported that Spike shares immunogenic linear motifs with, among others, thrombopoietin (TQPLL) and tropomyosin alpha-3 (ELDKY) [29]. These findings appear relevant since the former is a key growth factor required for megakaryocytic differentiation and platelet production, and the latter is a structural component of cardiomyocytes. In another study, it was reported that Spike shares 41 minimal immune determinants with 27 human proteins specific to the female reproductive system relating to oogenesis, uterine receptivity, decidualization, and placentation [30].

Clinical studies provided evidence that the injection of COVID-19 mRNA vaccines can be associated with the production of autoantibodies, i.e., non-anti-Spike antibodies recognizing self-antigens, as a possible consequence of general immune dysregulation. For instance, Xu and colleagues [31] found neutralizing anti-type I interferon antibodies in 10% of healthy vaccinated individuals, although with a limited sample size. In another study, 18% of patients developing PCVS have been found to produce autoantibodies against neurofilament subunits [32]. Even if, in some instances, autoantibodies may represent innocent bystanders, it is still unclear whether vaccination re-activates latent, pre-existing autoimmunity or induces the “de novo” generation of autoantibodies.

**Figure 2:**
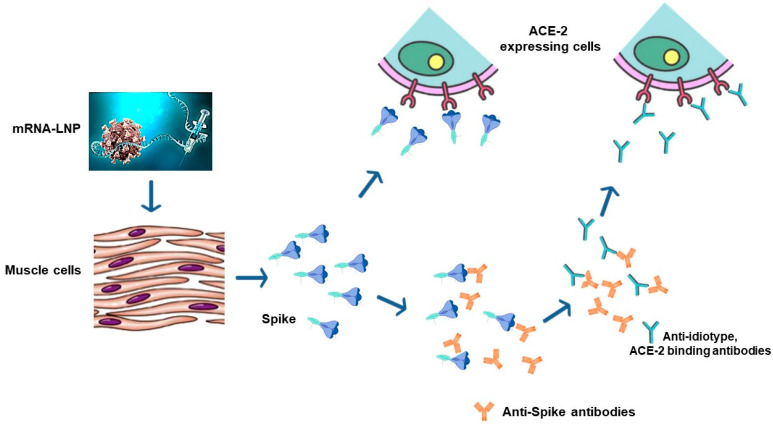
Generation of anti-idiotype antibodies after COVID-19 vaccination. The immune system can generate antibodies against the sequences of anti-Spike antibodies recognizing the Spike domain binding the ACE-2 receptor (receptor-binding domain, RBD). Through a mechanism of molecular mimicry, these antibodies (anti-idiotype antibodies) can bind ACE-2 just like the immunogenic Spike.

Molecular mimicry is also the basis of the effects of anti-idiotype antibodies (Figure 2).

In the case that the immunogen is an antigen binding to a molecular partner, the immune system can react against the sequences within the induced anti-antigen antibodies that recognize the region of the antigen that binds its partner, e.g., in the case of Spike, the receptor-binding domain (RBD). Under physiologic conditions, this mechanism contributes to the control of the production of antigen-specific antibodies. However, in the presence of exceeding amounts of antigen-specific antibodies, as in the case of mRNA-based anti-COVID-19 vaccination, the consequent hyper-production of anti-idiotype antibodies can lead to effects mimicking those induced by the binding of Spike with ACE-2 [33]. Bellucci and colleagues have recently demonstrated the side effects associated with the production of ACE-2-binding anti-idiotype antibodies. In particular, they reported neurological clinical complications including radiculitis, myelitis, and Guillain–Barré syndrome in both SARS-CoV-2-infected and uninfected subjects injected with mRNA-based COVID-19 vaccines and developing anti-ACE-2 autoantibodies [34]. Regrettably, both autoantibodies and anti-idiotype antibodies are expected to persist beyond the duration of the anti-Spike immune response.

The recent discovery that the incorporation of N1-methyl-pseudouridine in place of the natural uridine residue in the backbone of vaccine-associated mRNA can induce a +1 ribosomal frameshifting added another layer of complexity in terms of the immune response induced by the vaccine. It was estimated that roughly 8% of the total translated products represent unknown proteins that are immunogenic in humans [35]. The autoimmune potential of the aberrant protein products generated in this way represents an additional point that must be investigated further in depth.

## 6. Mucosal Vaccines: An Alternative Potentially Free of Systemic Side Effects

The COVID-19 battlefield is the respiratory system, where the ideal COVID-19 vaccine should develop its most effective immunologic and antiviral strength. Clinical data reported regarding current mRNA-based COVID-19 vaccines support the idea that the strong circulatory immune response is associated with antiviral immunity in the respiratory districts that is too limited [36].

Similarly to what has been demonstrated with natural infections [37], mucosal vaccines have the potential to elicit effective immune responses in the respiratory compartment through the induction of both neutralizing dimeric/secretory IgAs in the oronasopharingeal district [38], and antiviral resident memory CD8^+^ T lymphocytes in the lower respiratory tract [39]. In this way, effective mucosal vaccines have the incomparable advantage of blocking the transmission chain of SARS-CoV-2 as well as other airborne viruses.

At present, two COVID-19 mucosal vaccines have been approved, and others are in clinical experimentation [40]. Of note, in no cases are these vaccines expected to induce robust systemic immune responses like those observed with current COVID-19 vaccines. However, suboptimal/weak systemic immunization should not be considered a functionally relevant disadvantage considering the compartmentalization of the respiratory immune system [41], which limits the access of neutralizing IgGs and antiviral immune cells from the circulatory district. Conversely, it represents an advantage in terms of a strong reduction in/lack of immunologic systemic effects induced by parenterally injected mRNA-based COVID-19 vaccines, including the production of undesirable circulatory anti-idiotypic antibodies.

## 7. Conclusions

Several experimental pieces of evidence support the idea that the Spike protein is produced abundantly and persists after mRNA COVID-19 vaccination. However, current mRNA-based COVID-19 vaccines recognize a series of relevant limitations including the rapid waning of the immune response, the inability to mount an effective immune response at the virus port of entry, and the reduced efficacy of updated formulations due to the phenomenon of original antigenic sin [42,43]. On the other hand, powerful mRNA translation coupled with Spike overproduction can lead to the dysregulation of ACE-2 signaling and cytokine production, antibody cross-reaction against unspecific molecular targets, the emersion of both auto- and anti-idiotype antibodies, and immune responses of uncertain significance against unknown products. In addition, the cytokines produced after Spike/ACE-2 binding can unfavorably influence the fate of still “dormant” tumors and pre-existent autoimmune pathologies as well as chronic inflammation. For these reasons, the current indication of COVID-19 mRNA vaccines for the “fragile” population should be carefully re-evaluated in light of the typology of each specific fragility.

Notwithstanding the remarkable efficiency of antigen production, attempts to ameliorate the performance of these mRNA-based COVID-19 vaccines have been made in the direction of enforcing Spike production through the parenteral injection of self-replicating mRNA-based vectors [44]. Notably, the Japanese Ministry of Health has recently approved a clinical trial for testing the safety and effectiveness of a COVID-19 vaccine based on this technology [45]. This choice appears to be truly questionable given the above-described shortcomings induced by the exceeding production and persistence of circulatory Spike dictated by current mRNA-based COVID-19 vaccines. In this scenario, increasing the amounts and the persistence of circulating Spike is expected to exacerbate both cellular and immunologic side effects, but without acting on the most relevant functional limitation of these vaccines, i.e., their inability to elicit neutralizing immunity in the respiratory tracts due to the immune compartmentalization of the respiratory system. In addition, a too-potent and persistent immunogenic stimulus is known to induce immunologic tolerance, as also reported in a couple of papers for current COVID-19 vaccines [46,47].

Conversely, a more plausible avenue to be paced is represented by the development of effective mucosal vaccines [48] given their ability to act at the virus port of entry and to avoid most of the systemic side effects observed in intramuscularly injected COVID-19 mRNA vaccines.

mRNA-based technology is currently attracting the interest of many scientists worldwide. In the case of COVID-19 vaccines, it seems more than reasonable that an adequate burden of investigations would be focused on the identification and analysis of unexpected events, with the obvious intent to render this prophylactic strategy safer and commensurate for use in a large number of healthy people.

## Data Availability

No new data have been created.
